# Heparin and Heparin-Derivatives in Post-Subarachnoid Hemorrhage Brain Injury: A Multimodal Therapy for a Multimodal Disease

**DOI:** 10.3390/molecules22050724

**Published:** 2017-05-02

**Authors:** Erik G. Hayman, Akil P. Patel, Robert F. James, J. Marc Simard

**Affiliations:** 1 Department of Neurosurgery, University of Maryland School of Medicine, Baltimore, MD 21201, USA; ehayman@som.umaryland.edu (E.G.H.); appatel@som.umaryland.edu (A.P.P.); 2 Department of Neurosurgery, University of Louisville, Louisville, KY 40208, USA; robert.james@louisville.edu

**Keywords:** heparin, enoxaparin, subarachnoid hemorrhage, edema, brain injury, inflammation

## Abstract

Pharmacologic efforts to improve outcomes following aneurysmal subarachnoid hemorrhage (aSAH) remain disappointing, likely owing to the complex nature of post-hemorrhage brain injury. Previous work suggests that heparin, due to the multimodal nature of its actions, reduces the incidence of clinical vasospasm and delayed cerebral ischemia that accompany the disease. This narrative review examines how heparin may mitigate the non-vasospastic pathological aspects of aSAH, particularly those related to neuroinflammation. Following a brief review of early brain injury in aSAH and heparin’s general pharmacology, we discuss potential mechanistic roles of heparin therapy in treating post-aSAH inflammatory injury. These roles include reducing ischemia-reperfusion injury, preventing leukocyte extravasation, modulating phagocyte activation, countering oxidative stress, and correcting blood-brain barrier dysfunction. Following a discussion of evidence to support these mechanistic roles, we provide a brief discussion of potential complications of heparin usage in aSAH. Our review suggests that heparin’s use in aSAH is not only safe, but effectively addresses a number of pathologies initiated by aSAH.

## 1. Introduction

Despite decades of research, aneurysmal subarachnoid hemorrhage (aSAH) significantly compromises quality of life in those patients who survive their initial hemorrhage. Half of all surviving patients demonstrate some significant deficit of language, memory, or executive function [1,2,3,4], with only a third of survivors ultimately returning to work [5]. Consistent with these clinical findings, radiographic evaluation of survivors of aSAH demonstrates significant degrees of brain atrophy and other signs of global brain injury [6,7]. Although infarction due to vasospasm undoubtedly explains the acquisition of new focal deficits following aSAH, vasospasm-centered theories do not compellingly account for this global brain injury, especially given that effective pharmacologic prevention of vasospasm fails to improve outcomes following aSAH [8].

Modern understandings of post-SAH brain injury recognize the importance of other pathophysiological processes [9,10] emphasizing inflammation in particular as a central component of post-SAH brain injury [11]. SAH generates a wide variety of hemoglobin breakdown products capable of wide dissemination via the CSF, leading to global inflammation via activation of receptors such as toll-like receptor 4 [12,13], attraction of inflammatory phagocytes [14], and creation of a pro-oxidative environment via depletion of anti-oxidants [15]. Several clinical studies link this systemic inflammatory response to brain injury and poor outcome following aSAH [7,16,17,18,19]. Therefore, mitigating this inflammatory response presents an attractive therapeutic approach to improving outcomes following aSAH. Although specific inhibition of pro-inflammatory regulators, such as cytokines, represents a valid therapeutic strategy [20], aSAH activates multiple inflammatory pathways, suggesting that blockade of any single upstream inflammatory initiator may not adequately curb the downstream inflammatory response.

Multiple lines of clinical and pre-clinical evidence suggest that the endogenous anti-coagulant heparin effectively reduces inflammation and improves outcomes following aSAH [21,22,23,24]. Perhaps because of its clinical use as an anti-coagulant and its widely studied interactions with the coagulation cascade, clinicians remain relatively ignorant heparin’s promiscuous interaction with a number of biological processes, especially those involved with inflammation. Although this absence of specificity typically hinders the application of drugs like heparin as therapeutic agents, these pleiotropic effects may prove advantageous in the complex inflammatory milieu accompanying aSAH. This review seeks to summarize the potential benefits of heparin therapy in the context of aSAH, with a particular emphasis on its anti-inflammatory properties.

## 2. Early Brain Injury, Inflammation, and Blood-Brain Barrier Failure in Aneurysmal Subarachnoid Hemorrhage

Aneurysm rupture initiates two separate pathologic events that contribute to global brain injury. The initial rupture rapidly raises intracranial pressure, leading to cerebral hypoperfusion, possible cerebral circulatory arrest, and global brain injury analogous to that of cardiac arrest [25]. The second occurs gradually in the days following hemorrhage as the subarachnoid blood clot breaks down, releasing a variety of hemoglobin and coagulation by-products into the CSF, where they undergo global distribution. Although these events negatively affect nearly every aspect of the CNS, they have a number of specific consequences for the brain, of which vasospasm represents only a single component.

SAH induces a significant degree of CNS inflammation, both from activation of the brain’s endogenous microglia as well as influx of circulating leukocytes [26,27,28,29,30]. Inflammatory activation of these cells has a number of deleterious effects. Activated microglia mediate the significant neuronal apoptosis observed following experimental SAH [26]. Brain invasion by circulating neutrophils induces microvascular dysfunction [31], endothelial injury [32], and memory deficits via NMDA receptor dysfunction [29]. Perivascular invasion by leukocytes plays a role in vasospasm, as blockade of invasion via monoclonal antibody prevents vasospasm in both rabbits [33] and monkeys [34]. Generation of reactive species such as peroxynitrite via phagocyte-derived inducible nitric oxide synthase contributes to oxidative brain injury following aSAH [35]. This inflammatory state following aSAH mediates brain injury over several weeks [36]. Despite its clear role in brain injury following aSAH, this inflammatory response is not entirely detrimental. Both endogenous and circulating phagocytes mediate hemoglobin clearance following hemorrhage [37,38]. Activated microglia and their inflammatory cytokines also promote neurogenesis in non-aSAH models via activation of the brain’s stem cell compartment [39,40], a relevant finding given evidence of increased neurogenesis following human aSAH [41]. However, despite these potentially beneficial effects, the excess inflammation that follows aSAH causes more harm than benefit, especially in the early post-ictal period.

Besides directly modulating the CNS microenvironment, aSAH induces failure of the blood brain barrier with consequent vasogenic edema formation. Diverse mechanisms underlie this phenomenon, as ischemia-hypoperfusion injury and hemorrhagic products within the subarachnoid space both contribute to blood-brain barrier (BBB) dysfunction following aSAH [42,43,44]. Besides brain swelling due to vasogenic edema [45], BBB dysfunction contributes to several other pathologic processes. Perfusion studies in patients with delayed cerebral ischemia following aSAH demonstrate abnormal permeability of the BBB prior to actual infarction [46]. Experimental studies link the loss of white matter integrity to BBB disruption following SAH [47]. Finally, the BBB represents a crucial gateway for inflammatory cell infiltration of the brain [48]; loss of barrier integrity could augment this already significant neuroinflammatory response to aSAH.

## 3. Heparin—Physiologic and Pharmacologic Roles

The heparins refer to a class of endogenous glycosaminoglycans originally isolated from canine liver cells during investigations of endogenous coagulants (hence, the Greek root *hepar* meaning “liver”) [49]. Heparin molecules consist of linear polymeric chains of heavily sulfated polysaccharide chains. This high degree of sulfation imparts the highest negative charge density of any known biological macromolecule, allowing heparin to interact with a large number of proteins [50]. Given its context of discovery as well as its utility as an anticoagulant, traditional understandings of heparin’s pharmacologic actions situate it within the clotting cascade. Within this understanding, heparin’s primary role is to bind antithrombin III, inducing an allosteric activation of the latter that allows antithrombin III to inhibit the clotting Factor Xa, a phenomenon relatively independent of the size of the heparin molecule [51]. By virtue of heparin’s large size and high negative charge, the heparin-antithrombin III complex may also bind thrombin and inactivate thrombin, although this action crucially depends on sufficient chain length to bind both antithrombin III and thrombin. Low molecular weight heparins exploit this latter property to inhibit Factor Xa without appreciable anti-thrombin activity. Of note, the anti-coagulant properties of heparin depend on its negative charge to allow for both thrombin binding and allosteric activation of antithrombin III; stripping heparin molecules of their negatively charged sulfate moieties reduces heparin’s function as an anticoagulant [52].

Despite the prominence of anti-coagulation in heparin’s clinical applications, several lines of evidence suggest that anti-coagulation is not heparin’s primary physiologic role. Despite its initial discovery in mammals, heparin-like molecules exist in a wide variety of species lacking a formal hematologic system, including arthropods and echinoderms [53]. Furthermore, within mammals, mast cells and basophils represent the most abundant histologic source of heparin [54]; the importance of these cells in allergic and anti-helminthic response would seem to situate heparin within the broader schema of type 2 immune responses [55]. Finally, besides well-characterized interactions with antithrombin III, heparin promiscuously interacts with a variety of inflammatory proteins and chemokines [56]. While heparin’s anticoagulant properties undoubtedly play a role in inflammatory physiology, a simple understanding of heparin as anticoagulant neglects heparin’s broader role as a modulator of inflammatory function.

Heparin and its derivative molecules possess significant anti-inflammatory activity [57]. Heparin and heparinoids exert their anti-inflammatory effects through a wide variety of mechanisms. At the level of signal transduction, they reduce LPS-induced nuclear translocation of NF-κβ and the associated inflammatory response both in vitro and in vivo, although mechanistic explanations of this phenomenon are lacking [58,59]. Heparin molecules bind and inhibit cellular adhesion molecules such as the selectins [60,61], thereby preventing lymphocyte homing and extravasation. Heparin binds with high affinity to a number of inflammatory cytokines including IL-12 [62] and IL-2 [63], potentially sequestering them. Heparin prevents activation of the effector cells of inflammation, such as neutrophils and macrophages [64,65]. Finally, heparin directly inhibits molecular mediators of inflammation, such as elastase [66] and major basic protein [67]. Compellingly, the anti-inflammatory actions of heparin may be independent of its anti-coagulant activity [68]. Given its potent role as an anti-inflammatory agent, several clinical trials have evaluated heparin and its derivatives in diseases of inappropriate inflammation, including sepsis [69,70], and asthma [71].

Heparin’s main clinical roles in the management of aSAH, prevention of VTE and systemic heparinization during endovascular treatment of aneurysms, exploit its anticoagulant properties. However, the broader role of heparin outside the coagulation cascade argues for a broader utility in aneurysmal SAH. Several clinical studies already suggest that heparin and its low molecular weight derivative enoxaparin reduce the incidence of clinical vasospasm and delayed cerebral infarction following aneurysmal SAH [21,24,72], although the literature is not entirely consistent in this result [73]; a previous review already discussed the role of heparin in the prevention of delayed cerebral ischemia following aneurysmal SAH [74]. Beyond vasospasm, however, heparin may improve outcomes in aSAH by preventing inflammation and restoring blood-brain barrier integrity. Given the increasing recognition of inflammation, edema, and blood-brain barrier dysfunction as mediators of poor outcome following aSAH, these extravascular effects of heparin represent an even more important aspect of its therapeutic efficacy than the prevention of vasospasm.

## 4. Heparin in Post-SAH Brain Injury

### 4.1. Ischemia-Reperfusion Injury

A hallmark of aneurysm rupture is a transient period of hypoperfusion or even frank intracranial circulatory arrest secondary to the acute rise in intracranial pressure at ictus. Although other factors, such as CSF dissemination of blood products, undoubtedly play a role in secondary brain injury, the ischemia-reperfusion injury at ictus is arguably the predominant injury following aSAH, as its clinical correlate, loss of consciousness at ictus, remains one of the best predictors of outcome following aSAH [75,76]. Multiple studies demonstrate the efficacy of heparin and its derivatives in ischemia-reperfusion injury. Several studies in rats demonstrate reduced infarct volume following heparin and heparin-derivative administration following transient cerebral arterial occlusion [77,78,79,80,81]. Both the efficacy of non-anticoagulating 2,3-*O*-desulfated heparin derivative in vivo [79] as well as the neuroprotection low molecular weight heparin affords against ischemia-reperfusion injury in vitro [82] suggest that heparin and its derivatives exert these effects independently of their actions on the coagulation cascade. Proposed modes of action include neutralization of oxidative species [83] and inhibition of neuronal apoptosis [84], although neither of these studies provide definitive evidence of these modes of action.

### 4.2. Leukocyte Extravasation

Mobilization of leukocytes from the periphery into inflamed tissue (leukocyte extravasation) is a crucial component of inflammation generally and post-SAH neuroinflammation specifically [85]. Administration of low dose heparin following experimental SAH reduces the number of inflammatory cells within the CNS [22]. Although studies of heparin in SAH do not address leukocyte extravasation explicitly, several animal models of neuroinflammatory injury suggest that heparins directly inhibit this process. Administration of unfractionated heparin following murine TBI reduces leukocyte extravasation as demonstrated by in vivo microscopy [86]. Administration of low molecular weight heparin yields similar results [87]. Compellingly, an in vivo microscopy study of experimental meningitis also demonstrates a reduction in leukocyte extravasation with heparin administration, a phenomenon attributable to both a reduction in leukocyte sticking and leukocyte rolling to the endothelium [88]. These findings of reduced leukocyte adhesion and leukocyte extravasation following heparin administration in disparate neuroinflammatory conditions suggests a common mechanism of action. Although the movement of leukocytes from the vascular to the parenchymal compartment is a complex process, adhesion of leukocytes to the vessel wall critically depends on interactions between cell surface glycoproteins and selectins, surface lectins expressed by both leukocytes (L-selectin) and endothelial cells (E-selectin) [89]. Given current knowledge of heparin’s effects on endothelium-leukocyte interactions, inhibition of leukocyte expressed selectin [90] provides a plausible candidate mechanism to explain its effects on leukocyte extravasation. Several studies demonstrate that heparin-mediated inhibition of selectins occurs independently of anti-coagulation [60,91]. Consistent with these observations, studies of heparin in experimental TBI [86] note equivalent efficacy of low, non-anticoagulating heparin doses compared to higher doses with regard to leukocyte extravasation.

### 4.3. Inflammatory Activation

Although heparin’s effects on leukocyte extravasation mediate some aspects of its anti-inflammatory processes, heparin may also reduce injury by modulating inflammatory cell activation. A key effector mechanism in the pathological neuroinflammatory response following SAH is phagocyte activation, resulting in production of direct mediators of pathology such as peroxynitrite [35] and matrix metalloproteinase-9 [47]. Given that heparin’s effects on leukocyte extravasation do not readily account for the reduction in endogenous microglial activation observed with heparinization following SAH [22], heparin’s effects on activation of inflammatory cells appear relevant to SAH. Given heparin’s wide array of interactions with inflammatory mediators, no single mechanism of heparin induced immunoquiescence likely accounts for its full pleiotropic effects. Nevertheless, several interesting candidate mechanisms bear further discussion.

The receptor for advanced glycation end-products (RAGE) expressed on phagocytes mediates a number of inflammatory effects via activation of NF-κB [92]; SAH induces RAGE expression throughout the cortex by both neurons and microglia [93]. Furthermore, SAH induces the expression and release of a number of RAGE ligands, including HMGB1 [93,94,95,96,97,98] and S100B [99,100,101,102], within the CNS. Consistent with its inflammatory role, inhibition of RAGE signaling reduces inflammation and improves functional outcomes following experimental SAH [103,104,105]. Heparins, including low molecular weight heparins and non-anticoagulating derivatives such as 2,3-*O*-desulfated heparin, inhibit interactions between RAGE and its ligands with a high degree of specificity [65,106,107,108,109,110]. Although not well studied in SAH, pharmacologic blockade of HMGB1 duplicates some of the beneficial effects of enoxaparin following experimental TBI, including reduction of leukocyte extravasation, suggesting that heparin mediates some of its effects via modulating RAGE’s interactions with its ligands [111]. Blockade of RAGE inhibition may account for some of heparin’s effects on inflammatory cell activation.

Another potential mechanism of inflammatory modulation focuses on macrophage polarization. Although overly simplistic, macrophages and microglia roughly partition into two distinct phenotypes [112]. The M1 phenotype promotes anti-cellular immune responses important to viral and tumor defense. Consistent with this function, M1 phagocytes mediate a variety of biological effects relevant to post-SAH brain injury including peroxynitrite production via upregulation of inducible nitric oxide synthase and production of inflammatory cytokines including IL-1β, processes that contribute to early brain injury following SAH [35,113]. M2 phagocytes, in contrast, have opposite effects, reducing nitric oxide via arginine consumption and producing anti-inflammatory cytokines. Modulation of phagocyte polarization from a M1 to a M2 phenotype appears protective in SAH [114]. As mentioned previously, mast cells and basophils are the predominant sources of physiologic heparin [115]. This association strongly suggests that heparin plays a role in allergic and anti-helminthic responses. A common murine marker of M2 phenotype, the Ym1 receptor appears necessary for generation of a Type II immune response [116,117]. Compellingly, heparin appears to be a physiologic ligand of Ym1 [118]. Although direct evaluations of heparin’s physiologic effects on phagocyte polarization are lacking, a study of oral enoxaparin in experimental ulcerative colitis demonstrates modulation of colonic macrophages from an M1 to an M2 phenotype with enoxaparin administration [119], a finding consistent with this hypothesized physiologic role. Further studies are required, however, to establish whether heparin modulates phagocyte polarization in aSAH.

### 4.4. Oxidative Stress

Production of reactive oxidative species (ROS) by activated phagocytes mediates significant injury following SAH [35]. Strategies to reduce oxidative stress following experimental SAH demonstrate numerous beneficial effects [120,121,122,123]. Heparin demonstrates a number of anti-oxidant effects. Extracellular superoxide dismutase (EC-SOD), a powerful anti-oxidant enzyme, demonstrates specific heparin binding [124,125]. From a physiologic standpoint, binding of EC-SOD to cell surface heparan sulfate sequesters EC-SOD to the cell surface, as mutations in the heparin-binding domain of SOD or administration of IV heparin results in release of EC-SOD into circulating fluids [126,127]. Aside from increasing circulating levels of EC-SOD, heparin also appears to induce its synthesis [128]. This release of EC-SOD into circulating fluids increases its activity, as heparin administration into the CSF of rabbits overexpressing EC-SOD results in a 27-fold increase in SOD activity within the CSF [129]. Although not studied in subarachnoid hemorrhage, systemic administration of enoxaparin enhances brain SOD activity in experimental cerebral ischemia-reperfusion injury [83], confirming the relevance of this phenomenon to intracranial pathology. Thioredoxin reductase, another enzyme thought protective in oxidative brain injury [130,131], also demonstrates high affinity binding to heparin [132], although the significance of this interaction is unclear. Several in vitro studies demonstrate direct antioxidant actions of the heparin molecule itself independent of any associated co-enzyme [133,134]. Taken together, these findings suggest that heparin may ameliorate the oxidative injury generated following aSAH.

### 4.5. Blood-Brain Barrier Dysfunction and Vasogenic Edema

Edema formation following SAH portends a poor prognosis [42]. Blood-brain barrier dysfunction and its associated vasogenic edema therefore present highly plausible therapeutic targets in aSAH. Multiple lines of evidence, in both aSAH and other injuries, suggest that heparin reduces blood-brain barrier dysfunction and its associated vasogenic edema. In a murine endovascular perforation model of SAH, pretreatment with low dose heparin reduced edema formation at 24 h following injury, with improved early behavioral outcome [23]. Heparin administration in other forms of experimental brain injury reduces blood-brain barrier dysfunction and edema formation in models as diverse as TBI [86,87,111,135], ischemic stroke [78,136], intracerebral hemorrhage [137,138], and meningitis [88]. A human study of TBI patients corroborates these findings, demonstrating more rapid resolution of pathologic CT imaging features, especially edema, with early enoxaparin administration [139].

Given the intimate association between inflammation and edema, heparin’s anti-edema effects most likely derive from its anti-inflammatory properties. However, heparin demonstrates significant interaction with two molecules relevant to edema formation following aSAH, vascular endothelial growth factor (VEGF) and bradykinin (BK) [42]. VEGF, an angiogenic protein initially identified as a vascular permeability factor, binds heparin [140]. The effects of heparin on VEGF-endothelial cell interactions are complex. At low concentrations, heparin appears to enhance binding of VEGF to its receptor; however, at progressively higher concentrations heparin inhibits VEGF binding [141]. This inhibitory effect is sensitive to both the sulfation [141] and the size of heparin molecules employed [142]. Given the role of VEGF in SAH-mediated edema specifically and brain edema generally, heparin inhibition of VEGF presents a plausible albeit uninvestigated mode of edema reduction. Like VEGF, the interactions between heparin and bradykinin appear complex. While mast cell-derived heparin is crucial to both bradykinin formation and vascular permeability in allergic conditions [143], other studies suggest that intravenous heparin inhibits bradykinin induced vascular permeability [144]. Furthermore, bradykinin-mediated edema in aSAH occurs very early following hemorrhage [145]. Taken together, these findings suggests that heparin’s interactions with bradykinin may not be relevant to its effects on edema.

## 5. Complications of Heparin Therapy in aSAH

### 5.1. Heparin Induced Thrombocytopenia (HIT)

Heparin-induced thrombocytopenia is arguably the most feared heparin related complication. An autoimmune phenomenon, HIT results from antibody formation to the heparin-platelet factor 4 complex, resulting in both platelet depletion and a paradoxical pro-thrombotic state with significant risk of both arterial and venous thromboembolic complications [146]. Although studies suggest that 3 to 5 percent of patients undergoing intravenous unfractionated heparin will develop HIT, the incidence of symptomatic HIT following SAH may be much higher, with a reported incidence between 5% and 15% [147,148]. Although general studies associate LMWH with a reduced risk of symptomatic HIT, the one comparison available to date finds no difference in rates of HIT between LMWH and UFH in SA [148]. Despite its associated thrombocytopenia, ischemic complications represent a particular concern with symptomatic HIT. Several large series identify increased risk of stroke, death, and poor outcome in SAH patients with HIT [147,149]. Risk factors identified for HIT in a multivariate analysis of patients with aSAH included female gender, clip treatment of aneurysm, and greater number of vasospasm treatments [149]. This last association may reflect either increased risk of HIT due to use of catheter heparinization in the treatment of vasospasm or, alternatively, an increased risk of cerebral ischemia due to a HIT-induced pro-thrombotic state. In our own reported series of low dose infusion for aneurysmal SAH, none of the forty-three treated patients developed symptomatic HIT [21], despite use of clip treatment and prolonged (fourteen day) heparin exposure. This lower than expected incidence could reflect the use of a low dose (12 U/kg/h), reduced auto-antibody production due to heparin’s anti-inflammatory properties, a reduced incidence of vasospasm, or an inadequate number of subjects to assess HIT in this population. Since publication of this initial experience, two incidences of symptomatic HIT (2/150 patients) have developed in patients undergoing low dose heparin infusion, requiring cessation of their heparin infusion. Both patients required multiple interventions for vasospasm, with one experiencing a good recovery; the other died secondary to cerebral infarction. Although the low incidence of HIT in this population and excellent preliminary results of patients treated with low dose heparin outweigh this low risk of HIT, the apparently increased risk of cerebral ischemia from HIT in aSAH mandates vigilant screening for this condition.

### 5.2. Hemorrhagic Complications

Aside from HIT, fear of bleeding forms the other principal concern with heparin administration following aSAH. Patients with aSAH experience increased brain hemorrhage risk due to a variety of circumstances: aneurysm re-rupture prior to definitive treatment, periprocedural bleeding from surgery or a ventriculostomy, or hemorrhage into infarcted brain tissue. Although some experiences with systemic heparinization during endovascular treatment of ruptured aneurysms suggests that this practice does not increase the risk of hemorrhage or aneurysm re-bleeding [150,151], others suggest a significant risk of brain hemorrhage with this practice [152]. Ventriculostomy (EVD) placement followed by systemic heparinization for aSAH appears to be relatively safe, with multiple studies finding a low risk of significant EVD hemorrhage with systemic heparinization [151,153,154]. However, heparinization does appear to increase the risk of minor EVD hemorrhage [154]. In most reports of heparin or its derivatives as a therapeutic modality for vasospasm, hemorrhagic complications are either rare or non-existent [21,24,72], although one study noted an increased risk of non-significant bleeding in patients receiving enoxaparin compared to controls [73]. This low reported risk of hemorrhage is especially striking given the early (less than 12 h) post-operative initiation of heparin in at least one of these studies study [21]. The relatively low rate of hemorrhagic complications in these therapeutic studies of heparin most likely derive from a combination of low, non-anticoagulating doses of heparin as well as the relative safety of heparin use even following procedural interventions. Thus, the available evidence generally does not support the fears of hemorrhagic complications with heparinization following aneurysmal SAH.

## 6. Heparin Derivatives

**Figure 1:**
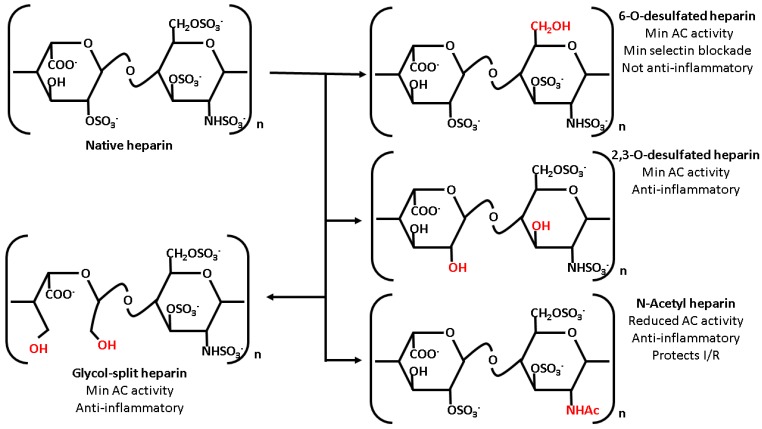
Summary of heparin chemical derivatives. Chemical modification of unfractionated heparin modulates its pharmacology. Although all derivatives discussed demonstrate reduced anticoagulant activity, chemical modification also affects heparin’s other pharmacologic properties in a regiospecific manner. 2,3-*O*-desulfated heparin demonstrates nearly retained selecting and RAGE inhibition; 6-*O*-desulfated heparin, despite numerous sulfated residues, fails to bind selectins and does not demonstrate significant anti-inflammatory effects in vivo. Although less well studied in inflammation, *N*-acetyl heparin does demonstrate evidence of efficacy in ischemia-reperfusion (I/R) injury despite reduced anticoagulant activity. Finally, glycol split heparin retains the ability to inhibit proteases such as elastase despite reduced anti-coagulant activity.

Although unfractionated heparin is perhaps the best studied member of the heparin family in the context of aSAH, its theoretical associated risk of hemorrhagic complications might preclude its use in certain patients, such as those with difficult to secure aneurysms. However, as briefly discussed earlier, heparin’s interactions with other molecular partners depends crucially on both the size and sulfation of the heparin molecule; modification of either of these parameters has significant effects on heparin’s pharmacologic effects. As previously discussed, the unfractionated heparin used clinically consists of polysaccharide polymers of significantly variable length. Cleavage of these polymers using either chemical or enzymatic digestion yields the low-molecular weight heparins. Low molecular weight heparins such as enoxaparin provide several advantages over standard unfractionated heparin, including more predictable clinical dosing and relatively specific anti-factor Xa activity [51]. Aside from digestion of unfractionated heparin, chemical synthesis of heparin’s anti-thrombin binding pentasaccharide motif yields fondaparinux, a potent anti-coagulant with highly specific inhibition of factor Xa without significant risk of heparin induced thrombocytopenia [155]. Aside from modification of polymer length, several other strategies change heparin’s pharmacological effects via chemical modification. Due to its high degree of sulfation, heparin possesses a significant high negative charge density, allowing for ionic interactions with a wide variety of molecular partners, including anti-thrombin III. Chemical desulfation reduces heparin’s anticoagulant properties while retaining its interactions with other molecular pathways. 2,3-*O*-desulfated heparin, prepared via cold alkaline hydrolysis of native heparin, demonstrates markedly reduced anti-thrombin III binding with a concurrent reduction in anti-coagulant activity while still retaining native heparin’s desirable anti-inflammatory effects such as selectin binding, RAGE blockade, and protease inhibition [108]. Replacement of the N-sulfate group with an acetyl side chain similarly reduces anticoagulant activity without compromising anti-inflammatory and anti-protease effects [156,157]. Although 6-*O*-desulfated heparin demonstrates reduced anti-coagulant activity with retained blockade of inflammatory receptors such as RAGE, loss of the 6-*O*-sulfate moiety also results in decreased selectin inhibition with compromised anti-inflammatory activity [158]. Finally, periodate cleavage of the 2,3 vicinal diols in uronate residues yield so-called glycol-split heparins, a class of heparin with reduced anticoagulant activity despite retained sulfate moieties. These glycol-split heparins demonstrate reduced anticoagulant activity while retaining useful features of native heparin, such as elastase inhibition and cytokine binding [157]. Although a full review of chemically modified heparinoids lies well-beyond the scope of this article, several of these molecules, especially 2,3-*O*-desulfated heparin, demonstrate a desirable blend of anti-inflammatory features with reduced anti-coagulant activity that might prove useful in aneurysmal SAH. Figure 1 depicts a summary of these modified heparin species along with salient characteristics.

## 7. Conclusions

**Figure 2:**
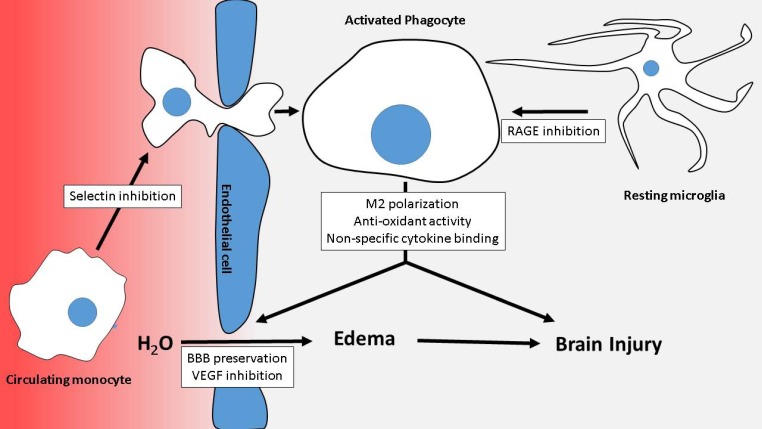
Summary of heparin’s modes of action. Several confluent processes combine to injure the brain including extravasation of circulating cells into the brain parenchyma, activation of microglia, production of harmful molecules and cytokines by activated phagocytes, and blood-brain barrier breakdown with subsequent vasogenic edema formation. Heparin antagonizes these processes, with heparin’s mechanisms of action indicated in the white boxes.

Given the complexity of brain injury following aneurysmal subarachnoid hemorrhage, no single therapeutic modality is likely to address all aspects relevant to its treatment. Given the multimodal nature of heparin’s interactions with mediators of post-SAH brain injury, as summarized in the accompanying figure (Figure 2), heparin and heparin derivatives may form a significantly better class of therapeutic for aSAH than more well-defined pharmacologic agents. Although initial clinical and experimental work suggests the efficacy of heparin in aSAH, these studies are relatively preliminary, with further clinical work required to confirm the utility of heparin. Furthermore, the absence of clear mechanistic insight into heparin’s therapeutic effects mandates further experimental work, as a better understanding of heparin’s mechanism of action could lead to improved and safer heparin derivatives. Nevertheless, heparin for aSAH represents a promising way forward in a disease much in need of effective therapy.
